# Biodegradable Nanoparticles Prepared from Chitosan and Casein for Delivery of Bioactive Polysaccharides

**DOI:** 10.3390/polym14142966

**Published:** 2022-07-21

**Authors:** Chi Lin, Fang-Yu Hsu, Wei-Ting Lin, Chia-Yun Cha, Yi-Cheng Ho, Fwu-Long Mi

**Affiliations:** 1 Graduate Institute of Medical Sciences, College of Medicine, Taipei Medical University, Taipei 11031, Taiwan; mervynlin11@gmail.com; 2 Department of Biochemistry and Molecular Cell Biology, School of Medicine, College of Medicine, Taipei Medical University, Taipei 11031, Taiwan; fangyu1004@gmail.com; 3 Department Oral and Maxillofacial Surgery, Ditmanson Medical Foundation Chia-Yi Christian Hospital, Chiayi 60004, Taiwan; ych07313@gmail.com; 4 Department of Bioagricultural Sciences, National Chiayi University, Chiayi 60004, Taiwan; trista881130@gmail.com; 5 Graduate Institute of Nanomedicine and Medical Engineering, College of Biomedical Engineering, Taipei Medical University, Taipei 11031, Taiwan

**Keywords:** chitosan, polysaccharides, bioactive and biodegradable, nanoparticles, pH-responsive, oral delivery, controlled release

## Abstract

*Ophiopogon japonicus* polysaccharides (OJPs) have great anti-inflammation and immunomodulatory abilities. However, the low bioavailability of OJPs reduces its applicability in the biomedical and pharmaceutical fields. Chitosan (CS) has excellent mucoadhesive properties and absorption-enhancing ability in oral administration. Casein hydrolysate (CL) has good interfacial diffusivity and emulsifying ability, and can interact with polysaccharides to form complexes combining the individual properties of both. Therefore, chitosan and casein hydrolysate are good candidates for developing nanoformulations for oral delivery. In this study, bioactive polysaccharides (OJPs), CS and CL, were combined to prepare CS/OJPs/CL co-assembled biodegradable nanoparticles. The interactions between polysaccharides (CS and OJPs) and peptide (CL) resulted in the formation of nanoparticles with an average particle size of 198 nm and high OJPs loading efficiency. The colloidal properties of the nanoparticles were pH-dependent, which were changed significantly in simulated digestive fluid at different pH values. OJPs released from the CS/OJPs/CL nanoparticles were greatly affected by pH and enzymatic degradation (trypsin and lysozyme). The nanoparticles were easily internalized by macrophages, thereby enhancing the OJPs’ inhibitory ability against Ni^2+^-induced cytotoxicity and LPS-induced nitric oxide production. This study demonstrates that prepared polysaccharide/protein co-assembled nanoparticles can be potential nanocarriers for the oral delivery of bioactive polysaccharides with anti-inflammatory functions.

## 1. Introduction

*Ophiopogon japonicus* is a traditional Chinese medicine that has been used for a long time in the treatment of cardiovascular and chronic inflammatory diseases, and has been proven to have anti-ischemic, anti-arrhythmic, anti-inflammatory, and microcirculation-improving effects [1,2,3,4,5,6,7,8,9,10]. The polysaccharides isolated from Ophiopogon japonicus (OJPs) have various biological activities, such as immunostimulation, anti-ischaemia, inhibiting platelets aggregation, and hypoglycemic [1,2,3,4,5,6,7,8,9,10,11,12]. However, the short half-life of OJPs and poor absorption after oral administration limit the efficacy and clinical application of OJPs. Therefore, a variety of drug delivery systems including erodible tablets, injectable in situ forming gels, nanoparticles, and liposome have been developed for controlled release and delivery of OJPs [13,14,15,16,17,18,19].

Chitosan (CS) is a biodegradable natural polymer consisting of D-glucosamine and N-acetyl-D-glucosamine residuals linked by β-(1,4) glycosidic linkage, which was obtained by the deacetylation of chitin. The chemical structure of CS is very similar to that of cellulose. CS is the only natural polysaccharide with cationic properties and has been widely used in the biomedical, pharmaceutical, cosmetic, and food industries. CS-based vehicles have been recognized as effective drug delivery systems for enhancing the oral bioavailability of drugs, phytochemicals and bioactive macromolecules including proteins, peptides and polysaccharides [20,21,22,23,24,25,26]. So far, many CS-based drug delivery systems such as hydrogels, microparticles, and nanoparticles (NPs) have been developed [20,27,28]. Among them, CS NPs are the most promising orally administered dosage forms, as they show great potential to enhance oral bioavailability [29,30,31,32]. The mucoadhesive properties allow CS NPs to easily attach to the mucus layer [26,33], thereby enhancing the gastrointestinal tract (GIT) residence time. Furthermore, the positive charges on CS NPs can transiently open tight junctions between epithelial cells to enhance drug permeation across the intestinal epithelium [34,35,36,37].

Compared with solvent evaporation induced phase separation and aldehyde cross-linking methods, polyelectrolyte complexing (PEC) method is widely used to produce CS NPs with tens to hundreds of nanometers in size under mild conditions. The electrostatic interactions between protonated CS and negatively charged polyanions such as alginate, fucoidan were responsible for the particle assembling under mild conditions [38,39,40]. Owing to the protonated and deprotonated states of the oppositely charged polyelectrolytes, these nanoparticles have pH-responsive properties with different characteristics of particle size, surface charge, and morphology at different pH conditions. These CS-based nanoparticles generally have excellent biodegradability and safety, and have functions such as controlled release of bioactive compounds, mucoadhesion, and intestinal permeability enhancement [40,41,42,43].

Casein (CA), the most abundant protein type in bovine milk, has a molecular weight of 19~25 kDa and an isoelectric point of 4.6~4.8. It mainly consists of four phosphoproteins (αS1-, αS2-, β-, and κ-CA), which shows amphiphilic properties that can form protein-polysaccharide complexes to exert an emulsifying effect [44,45,46]. Furthermore, the electronegative domains of CA are preferentially located in small peptide fragments, making these molecules susceptible to complex formation with cationic macromolecules [47,48]. CS is a cationic polymer that can form polyelectrolyte complexes with CA. CS/CA complex NPs have been developed for delivery of astaxanthin, anthocyanins, curcumin, fucoxanthin, platinum anticancer drug, and nattokinase [47,48,49,50,51,52,53,54,55], with the advantage of improved bioactivity and bioavailability, enhanced stability and water dispersibility, and sustained-release property.

Previously, we have isolated an OJP from the roots of *O. japonicus*. The OJPs are a group of anionic polysaccharides with molecular weight up to 27 kDa [56]. Due to the advantages mentioned above, this study aimed to develop nanocarriers for oral delivery of OJPs using CS and CA hydrolysate (CL). In this work, the anionic polysaccharides (OJPs) and cationic polysaccharide CS in aqueous solutions were self-assembled into nanoparticles via PEC method. Casein hydrolysate (CL)-based formulations are promising materials for stabilizing nanoemulsions [57]. Therefore, CL was incorporated into the CS/OJPs PEC NPs to form co-assembled CS/OJPs/CL NPs with increased stability. Furthermore, CL can be used to coat NPs to avoid premature drug release in the gastric environment and then can be specifically degraded by trypsin in the small intestine to trigger drug release [58,59]. The release properties of CS/OJPs/CL NPs were examined under different pH conditions and enzymatic degradation (lysozyme and trypsin). Furthermore, the protective effect of CS/OJPs/CL NPs against cytotoxicity of RAW264.7 cells induced by nickel, and the anti-inflammatory and free radical scavenging activities of the NPs were also evaluated. This is the first study using bioactive polysaccharides, chitosan, and peptides to prepare nanoparticles with pH- and enzyme-responsive properties and controlled release capability by a self-assembly method.

## 2. Materials and Methods

### 2.1. Materials

CS (MW = 80 kDa, DDAc = 85%) was purchased from Marine Bio Resources Co. (SSA190/301CF, Samut Sakhon, Thailand). CL (Hy-Case SF from bovine milk, C9386) and lysozyme (35,000 U/mg) was purchased from Sigma-Aldrich (St. Louis, MO, USA). Trypsin (2000 U/mg) was purchased from Gibco BRL (Paisley, UK).

### 2.2. Preparation of CS/OJPs/CL Co-Assembled Nanoparticles

OJPs was extracted and purified according to the method reported in our previous study [56]. The inversely charged CS, OJPs, and CL were employed to synthesize nanoparticles. This study prepared CS/OJPs/CL nanoparticles by co-assembling CS with OJPs and CL. CS was dissolved in 0.1M acetic acid aqueous solution (0.1% *w*/*v*) and CL was dissolved in water at pH 6.5 (0.2% *w*/*v*). OJPs (0.06% *w*/*v*), which represent an equivalent mass ratio of anionic polysaccharide, was dissolved in water. CS, OJPs, and CL solutions were mixed at CS:OJPs:CL volume ratio of 1.25:2.5:1.5 to obtain a CS:OJPs:CL weight ratio of 0.14:0.17:0.33 by magnetic stirring at room temperature to form CS/OJPs/CL co-assembled nanoparticles. CS/OJPs co-assembled nanoparticles were prepared as mentioned above by replacing CL with water.

### 2.3. Characterization of CS/OJPs/CL Nanoparticles

Particle size, zeta potential, and polydispersity index (PDI) of the test nanoparticles were analyzed using a Malvern Zetasizer (Nano-ZS, Malvern Instruments, Malvern, UK). Images were obtained after the test nanoparticle suspension was dropped onto carbon-coated copper grids and then allowing the solvent to evaporate. Shape and surface morphology of the nanoparticles were characterized by Hitachi H-600 TEM (Tokyo, Japan). The images were obtained after dropping the test nanoparticle suspension onto a carbon-coated copper grid and then allowing the solvent to evaporate. Chemical structures of the test nanoparticles were identified by Fourier transform infrared spectroscopy (Perkin Elmer FTIR Spectrometer Frontier, Waltham, MA, USA).

### 2.4. pH-Responsive and Biodegradable Properties

CS/OJPs/CL were placed in pH 3.0, 5.0, 6.5, and 7.4 buffer solutions to mimic the pH conditions of gastrointestinal tract, and particle size distribution (PSD) and polydispersity index (PDI) of the test sample solutions were analyzed over a predetermined period of time using a Malvern Zetasizer (Nano-ZS, Malvern Instruments, Malvern, UK) to evaluate the pH-responsive property of the NPs. Enzymatic degradation of the NPs was performed in PBS buffers containing 0.5 mg/mL of trypsin (2000 USP U/mg) and 1.0 mg/mL of lysozyme (35,000 U/mg), respectively. At a predetermined time interval, the biodegradable property of the NPs was investigated by determining PSD and PDI according to the method described above. Furthermore, during digestion, NPs samples were collected at different time points and their morphology was characterized by Hitachi H-600 TEM (Tokyo, Japan).

### 2.5. Drug Loading and Release

The unloaded OJPs were collected by using dialysis tubes Vivaspin^®^ 100 kDa MWCO (Hannover, Germany), and the remaining OJPs in the filtration was determined using a high-performance liquid chromatography (HPLC) (Varian ProStar Solvent Delivery System PS-210 and Varian ProStar 330 Photo Diode Array/PDA detector, Palo Alto, CA, USA). Drug encapsulation efficiency (EE%) were calculated according to the following Equation (1):

EE (%) = ((W_t_ − W_f_)/W_t_) × 100, (1)

where W_t_ was the weight of total OJPs added; W_f_ was the weight of free OJPs measured in the supernatant.

OJPs releases were investigated by placing nanoparticles in the 100 kDa MWCO dialysis tubing then immerse in release medium, including pH 6.5 buffers containing lysozyme (35,000 U/mL) or trypsin (1000 USP U/mL) with volume ratio NPs: medium = 1:9 at 37 °C. At predetermined time intervals, 400 μL of release medium was collected. The released OJPs were analyzed by using the previously mentioned HPLC method.

### 2.6. Cellular Uptake of Nanoparticles

FITC-labeled CS (FITC-CS) was synthesized according to the method reported in our previous study [56]. Afterwards, fluorescent CS/OJPs/CL NPs were prepared from FITC-CS, and then RAW 264.7 cells (4 × 10^4^ cells/well) were incubated with the FITC-labeled NPs (100 μg/mL) for 24 h. Cellular uptake of the FITC-labeled NPs by RAW 264.7 cells was visualized using a Leica TCS SP5 Spectral Confocal (Rotorua, New Zealand).

### 2.7. Cytotoxicity Assay

RAW 264.7 cells at a density of 1 × 10^4^ cells/mL were incubated with CS/OJPs/CL NPs (10, 50, and 100 μg/mL) for 24 h. Then, the medium was removed and the cells were treated with 100, 250, and 500 mΜ Ni^2+^ for further 24 h. After the incubation period, sulforhodamine B assay was performed to determine the viability of the Ni^2+^-treated cells by measuring the absorbance at 510 nm using a BioTek uQuant Microplate Reader (Winooski, VT, USA).

### 2.8. DPPH and ABTS Scavenging

The DPPH and ABTS radical scavenging activities of CS/OJPs/CL NPs were determined according to the method described in our previous study [56]. First, the CS/OJPs/CL NPs were sequentially diluted with water to prepare a series of sample solutions with different concentrations. Then, 270 μL of ABTS (7 mM ABTS/4.95 mM potassium persulfate) or DPPH (0.4 mM) reagent and 30 μL aliquots of each concentration of the sample solution were mixed in a 96-well microplate. After 30 min of reaction, the mixtures were diluted and the absorbances of the test samples were read at 517 nm (for DPPH assay) and 734 nm (for ABTS assay) using a BioTek uQuant Microplate Reader (Winooski, VT, USA).

### 2.9. Determination of Nitric Oxide

RAW 264.7 cells (1 × 10^4^ cells/mL) were co-cultured with CS/OJPs/CL NPs (5, 10, and 50 μg/mL) and then LPS was added to the macrophage cells at a final concentration of 10 ng/mL to stimulate inflammation. Nitric oxide (NO) produced by LPS-stimulated RAW 264.7 cells was measured by Griess assay. Briefly, the cell culture medium was mixed with an equal volume (100 µL) of a solution of Griess reagent, and incubated for 10 min at room temperature. The amount of nitrite (a stable metabolite of NO) in the supernatant was determined by measuring the absorbance at 540 nm using a BioTek uQuant Microplate Reader (Winooski, VT, USA).

### 2.10. Statistical Analysis

The experimental data were presented as the mean ± standard deviation (SD). Statistical evaluation among different study groups was analyzed by one-way ANOVA (*p* < 0.05 was considered statistically significant).

## 3. Results and Discussion

### 3.1. Optimization of CS/OJPs/CL Assembly

**Figure 1:**
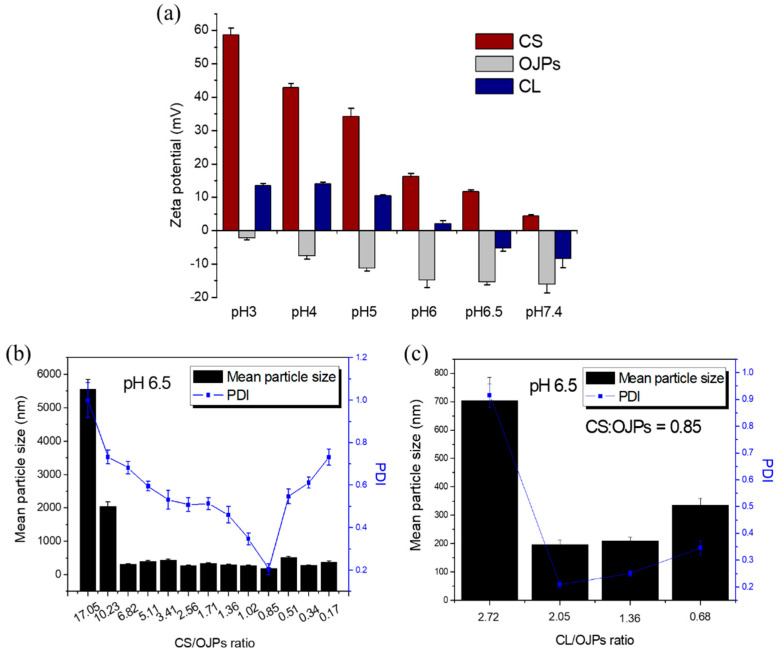
Formulation optimization: (**a**) zeta potential of CS, OJPs, and CL measured at different pH values, (**b**) mean particle size of CS/OJPs NPs prepare at different CS:OJPs mass ratio, (**c**) mean particle size of CS/OJPs/CL NPs prepare at different CL:OJPs mass ratio (CS:OJPs mass ratio = 0.85).

CS is a biopolymer with potential for oral or mucosal delivery of macromolecules, such as polypeptides, proteins, and polysaccharides. It has many advantages in drug delivery including mucoadhesion [26,39], enhanced penetration [34,35,36,37], and improved oral absorption [30,31]. It has also been used in the preparation of nanoparticles to control drug release and protect active compounds from enzymatic degradation and destruction by gastric acid [21,60]. In this study, CS, OJPs, and CL were combined to prepare nanoparticles for oral drug delivery. Figure 1a shows that at pH 6.5, the zeta potential of CS was positive but those of OJPs and CL were negative. Accordingly, CS/OJPs and CS/OJPs/CL NPs can be prepared by a polyelectrolyte complex method via electrostatic interactions between the oppositely charged polysaccharides and protein, which is a simple and mild method for preparation of nanoparticles. The average particle size and PDI of CS/OJPs NPs were mainly affected by the CS:OJPs weight ratio. The mean particle size greatly increased when the weight ratio was higher than 10, which might be attributed to the formation of NPs aggregates. In addition, the results of PDI and mean particle size analysis showed that the optimal preparation conditions for CS/OJPs NPs were at a CS:OJPs weight ratio of 0.85 (Figure 1b). A further increase or decrease in the weight ratio resulted in a significant increase in the PDI value, indicating that the particle size distribution of CS/OJPs NPs became non-uniform. Therefore, the CS:OJPs weight ratio of 0.85 was used for preparation of CS/OJPs/CL NPs.

Therefore, the driving force for the assembly of CS/OJPs/CL NPs is mainly attributed to the electrostatic attraction between negatively charged OJPs and CL and positively charged CS. Furthermore, there may be hydrophobic interactions between the hydrophobic residues of CS and CL. Previously, we have confirmed that the optimal preparation condition for CS/OJPs NPs is a CS:OJPs weight ratio of 0.85. When the CS:OJPs weight ratio was kept at 0.85, the preparation conditions for CS/OJPs/CL NPs were optimized by changing the OJPs/CL weight ratio. The results of PDI and mean particle size analysis showed that an OJPs:CL weight ratio of 2.05 was the optimal preparation condition (Figure 1c). Based on the preliminary analysis, the CS:OJPs:CL weight ratio of 0.14:0.17:0.33 was selected for production of CS/OJPs/CL NPs, since it exhibited the optimal particle size (198.1 nm) with a narrow size distribution (PDI = 0.21).

### 3.2. Characterizatio of OJPs/CS/CL NPs

**Figure 2:**
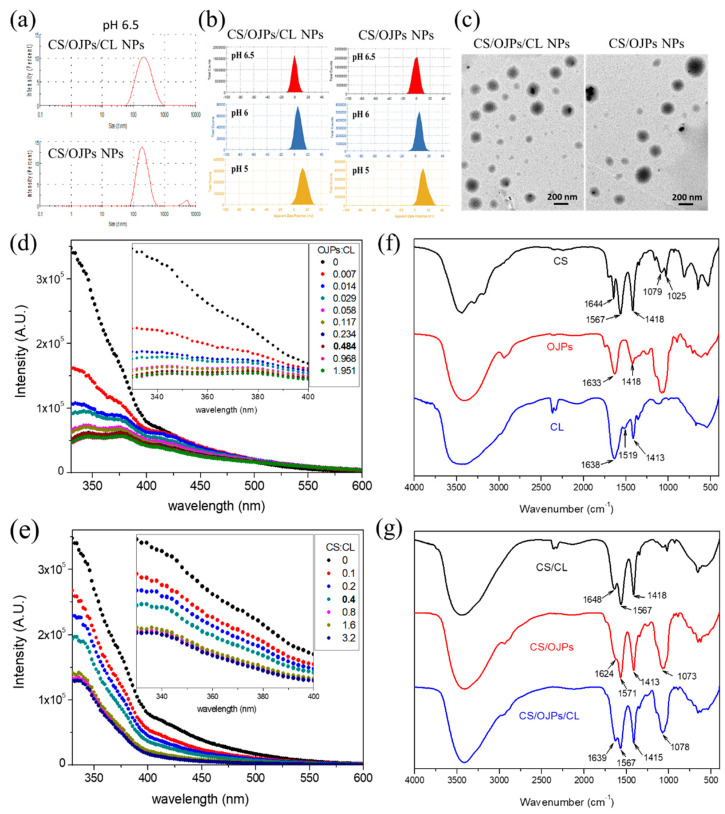
Characterization of CS/OJPs and CS/OJPs/CL NPs: (**a**) size distribution curve, (**b**) zeta potential curve, (**c**) TEM micrographs. Fluorescence spectra: (**d**) the mixtures prepared by adding OJPs to CL aqueous solution and (**e**) the mixtures prepared by adding CS to CL aqueous solution. FTIR spectra: (**f**) CS, OJPs and CL and (**g**) CS/OJPs and CS/OJPs/CL NPs.

Figure 2a shows the size distribution curves of CS/OJPs and CS/OJPs/CL NPs. The average size of both NPs was close to 200 nm (Table 1), but the size distribution curve of CS/OJPs showed a small peak in the range of 3000–6000 nm. This peak disappeared when CS/OJPs NPs was incorporated with CL, revealing that CL helped to increase the dispersion of nanoparticles to avoid aggregation. The zeta potential of CS/OJPs and CS/OJPs/CL NPs were positive (Figure 2b and Table 1), evidencing that the predominant cover on the particles’ surface is CS. However, as the value of pH increased from 5.0 to 6.5 (Figure 2b), the zeta potential of CS/OJPs and CS/OJPs/CL NPs decreased, which was due to the fact that the lower protonation degree of amino groups in CS but the higher ionization degree of carboxyl groups in OJPs. The results were consistent with pH-dependent changes in the zeta potential of CS, OJPs, and CL, respectively (Figure 1a). Instead of strong electrostatic interaction with negatively charged OJPs at pH 5.0, the NPs have slightly positive charges at pH 6.0 and 6.5, revealing that CS may have weaker electrostatic interaction with OJPs. TEM images show that both CS/OJPs and CS/OJPs/CL NPs have spherical shapes (Figure 2c).

The intrinsic fluorescence of tryptophan (Trp) and tyrosine (Tyr) is sensitive to conformational changes of protein molecules. The shift in their fluorescence wavelengths and fluorescence intensity can be used as an index to investigate the molecular interactions between CL and polysaccharides for assembly of nanoparticles [57]. The fluorescence emission spectra of CL, CL/CS, and CL/OJPs mixtures are shown in Figure 2d,e. The fluorescence emission maximum wavelength for CL was 350 nm. The CL emission peaks in the spectra of CS/CL and OJPs/CL mixtures were not obviously shifted but the fluorescence intensity of CL was quenched by addition of CS and OJPs solutions, respectively. The decrease in CL fluorescence intensity suggested the conformational change of CL due to specific interactions between CA and CS or OJPs, which led to quenching the fluorescence of CL by changing the π–π* transition of Trp and/or Tyr [52].

Figure 2f shows the IR spectra of CS, CL, and CS/OJPs and CS/OJPs/CL NPs. In the spectrum of CS, the strong absorption bands at 1567, 1644 and 3402 cm^−1^ were assigned to C=O stretching (amide I), N-H bending (amide II), and N-H and O-H stretching vibration. OJPs show strong absorption bands at 1633 cm^−1^, which was due to C=O stretching of carboxyl groups. Both CS and OJPs show the characteristic absorption bands of polysaccharides in the region of 1200–950 cm^−1^, which were assigned to pyranose ring vibrations (C-O-C and C-OH stretching). CL exhibited two major characteristic peaks for peptide bonds and, which were assigned to amide II absorption at 1519 cm^−1^ and amide I absorption at 1638 cm^−1^ [48]. The characteristic bands of OJPs in the spectra of CS/OJPs and CS/OJPs/CL NPs shifted from 1633 cm^−1^ to 1639 cm^−1^, which clearly indicated the presence of interactions between the oppositely charged polysaccharides (CS and OJPs) (Figure 2g).

### 3.3. pH-Responsive and Biodegradable Properties

Since the electrostatic interactions between the polyelectrolytes (CS, OJPs, and CL) are highly pH-dependent (Figure 1), the effect of pH on the stability of CS/OJPs/CL NPs needs to be investigated. Based on the oral and GIT routes of administration, nanocarriers must be able to resist multiple challenges including pH and gastric degradation. To mimic the environmental pH conditions upon oral intake of nanoformulations, in-vitro test media were prepared from different pH buffers representing fed stomach (pH 3.0), small intestine (pH 5.0–7.4). The size distribution and PDI of CS/OJPs/CL NPs in different pH media were measured to assess the pH-responsive property of the NPs [61].

**Figure 3:**
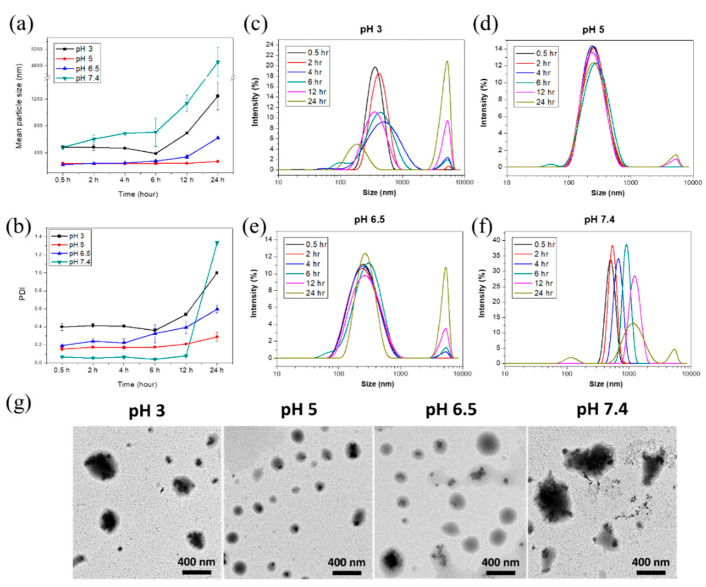
(**a**) Mean particle size, (**b**) PDI, (**c**–**f**) size distribution curves, and (**g**) TEM micrographs of CS/OJPs/CL NPs after 6 h at different pH conditions.

The CS/OJPs/CL NPs prepared in pH 5.0 and 6.5 buffers had a strong electrostatic interaction between the polysaccharides and protein hydrolysate, which led to more compact complexation that possessed smaller particle size. The hydrodynamic diameter of the NPs was 200–300 nm (Figure 3a), and the PDI and size distribution curves did not change significantly within 12 h (Figure 3b,d,e). As previously mentioned, the zeta potential of NPs in pH 5.0 buffer was 13.6 mV, indicating that positively charged CS dominates the particle surface (Figure 2b). At pH 6.5, the protonation degree of CS was reduced but it was still sufficient to provide electrostatic interactions with OJPs and CL, so the particle size did not increase significantly within 12 h. When the pH was increased to 7.4, the size increased to >4000 nm and the PDI increased to 1.4. At the pH value, OJPs and CL were negatively charged because their carboxyl groups were ionized but the amino groups of CS were deprotonated, resulting in reduced electrostatic interactions between CS and OJPs/CL. The NPs are readily disintegrated at pH 7.4, revealing that the NPs might release OJPs more rapidly at the distal ileum.

In pH 3.0 buffer, the hydrodynamic diameter of CS/OJPs/CL NPs was 480 nm (Figure 3a). CS was protonated while the carboxyl groups of OJPs and CLS tended to be in acid form rather than carboxylate ion, i.e., with hydrogen in the carboxylic acid groups, leading to a reduction in the electrostatic interactions between CS, OJPs, and CL. Notably, particle size and PDI increased significantly at 12 h. It is known that solid meals are usually emptied from the stomach within 3 to 4 h. Homogenized solid meals typically reduced digestion time in the stomach by 1–2 h. Generally, the recommended time for simulating gastric digestion of nanoformulations is 2 h. Based on the above findings, CS/OJPs/CL NPs may have the ability to prevent rapid drug release in the stomach before reaching the intestine.

Studies have shown CS/CA complex was stale in the pH range 4.0–6.0 [47]. To improve the stability of CS/CA-based nanoparticles (CS/CA NPs), several modifications have been performed, including using stearic acid-CS conjugate and CA [48], sinapic acid-grafted-CS and CA [53], and gallic acid-modified CS, CA and oxidized dextran [55], to fabricate the nanoparticles. These nanoparticles were stable over a wide pH range (2.0–7.4). Other studies enhanced the stability of CS/CA NPs by crosslinking of CS and CA with genipin and transglutaminase [50,54]. The NPs can enhance stability of curcumin and protect nattokinase from degradation by the acidic gastric juice. Our study shows that the OJPs, CS, and CA hydrolysate co-assembled NPs developed in this work are stable in the range of 3.0–6.5 without chemical modification and cross-linking. This may be because CL is a small peptide from CA hydrolysis, so it may have a stronger interaction with CS.

**Figure 4:**
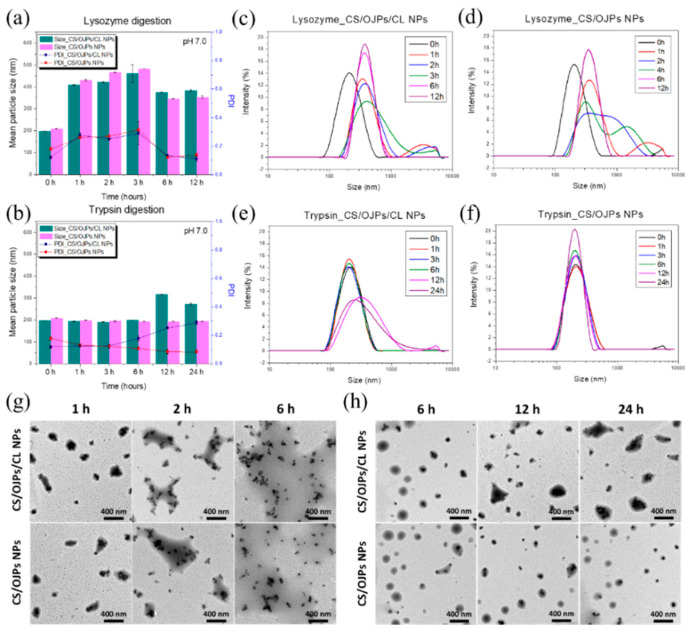
(**a**,**b**) Mean particle size and PDI of CS/OJPs and CS/OJPs/CL NPs degraded by lysozyme (1.0 mg/mL) and trypsin (0.5 mg/mL). (**c**,**d**) Size distribution curves of CS/OJPs and CS/OJPs/CL NPs degraded by lysozyme (1.0 mg/mL). (**e**,**f**) Size distribution curves of CS/OJPs and CS/OJPs/CL NPs degraded by trypsin (0.5 mg/mL). (**g**,**h**) TEM micrographs of CS/OJPs and CS/OJPs/CL NPs degraded by lysozyme (1.0 mg/mL) and trypsin (0.5 mg/mL).

The capability to maintain the integrity of CS/OJPs/CL NPs against enzymatic degradation was assessed by changes in size and PDI. As shown in Figure 4b, after incubation in simulated intestinal fluids (SIF) containing 0.5 mg/mL trypsin (pH 6.5), the particle size and PDI of CS/OJPs/CL NPs started to increase significantly at 12 h. The results suggested that the NPs can have the ability to protect drugs or proteins from enzymatic degradation by trypsin in intestinal juice within 6 h. In contrast, the particle size and PDI of CS/OJPs NPs did not change significantly compared to the initial values until 12 h; however, CS/OJPs/CL NPs changed, indicating that CL gives the NPs a trypsin response/degradation ability. Additionally, we examined the enzymatic degradability of CS/OJPs/CL and CS/OJPs NPs by lysozyme, which is generally abundant in mucosal surfaces. After 1 h incubation in SIF (pH 6.5) containing 1 mg/mL lysozyme, particle size and PDI of both NPs increased by more than two-fold. The high susceptibility of CS/OJPs/CL and CS/OJPs NPs to lysozyme hydrolysis can be attributed to the fact that these two NPs are mainly composed of CS.

### 3.4. Drug Loading and Release

**Figure 5:**
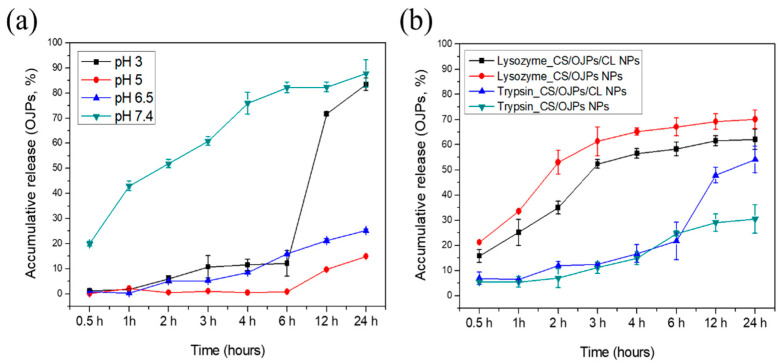
In vitro OJPs release from CS/OJPs and CS/OJPs/CL NPs: (**a**) OJPs release from the NPs at different pH conditions, (**b**) OJPs release from the NPs in pH 6.5 buffers containing lysozyme (1.0 mg/mL) and trypsin (0.5 mg/mL), respectively.

Nanoparticles need to have the ability to protect the loaded active compounds from degradation and premature release after ingestion, which is critical to ensure successful oral administration of drugs, proteins, and naturally occurring compounds. The encapsulation efficiency of OJPs in CS/OJPs and CS/OJPs/CL NPs were 78.6 ± 1.6% and 88.1 ± 0.2%. To examine whether the NPs are able to protect OJPs from premature release in the gastric environment and then successfully release OJPs in the small intestine, the simulated GI fluids were used to study the pH-dependent release properties of OJPs from the NPs. In vitro methods simulating the pH of the digestive tract are as follows: stomach (fasting—pH 2.0; fed—pH 3.0); duodenum (pH 4–6); proximal small intestine (pH 6.6); terminal ileum (pH 7.5). In Figure 5, the effects of CS/OJPs/CL and CS/OJPs NPs on the release of OJPs were investigated at pH 3.0, 5.0, 6.5, and 7.4. At pH 7.4, OJPs release from the NPs was fast. More than 75% of OJPs was released after 4 h in this medium, whereas there was almost no OJPs release at pH 5.0 and only 7.5% of OJPs were released at pH 6.5, respectively. This release pattern may be due to the fact that CS is deprotonated at pH 7.4 but OJPs and CL are negatively charged. The weakened electrostatic attraction and increased electrostatic repulsion between deprotonated CS and ionized OJPs and CL led to rapid decomposition of NPs and enhance the leakage of OJPs from the NPs into the releasing media. The amino groups in CS are protonated at pH 5.0 and 6.5, providing strong electrostatic attractions to the oppositely charged OJPs and CL. The release of OJPs from the NPs in both pH conditions showed a slow and sustained release manner. The results are consistent with results of previous particle size and PDI analysis that clearly showed that CS/OJPs/CL and CS/OJPs NPs were stable in the medium for 12 h.

To investigate whether CS/OJPs/CL NPs could prevent the premature release of OJPs in the stomach, the release behavior of the nanoformulation was tested in pH 3.0 buffer. In this release medium, OJPs release from CS/OJPs/CL NPs was slow during the first 6 h, followed by a quicker release of OJPs up to 24 h (≥80%). The electrostatic interaction between CS and OJPs were weakened because the carboxyl groups of OJPs were protonated. However, although CL carried net positive charge under low pH, negatively charged patches existing on CL micelle surface could still interact with chitosan [47]. Thus, these results indicated that CS/OJPs/CL NPs could be used to control OJPs release in acidic environment of the stomach and have potential for oral administration.

Studies have reported that genipin-crosslinked CS/CA NPs has the advantage of sustained release of curcumin at pH 7.4 [50], while genipin/transglutaminase co-crosslinked CS/CA NPs has the capability of controlled-release of anti-thrombotic drugs in the intestine [54]. Other studies show that L-arginine-functionalized CS/CA NPs released curcumin at a slower rate in simulated gastric fluid (SGF) but a faster rate in simulated intestinal fluid (SIF) [54]. These studies suggest that these nanocarriers with the ability to reduce gastric acid drug release can be used for the oral administration of small and macromolecular bioactive compounds and can improve their bioavailability. Our study shows that OJPs release from the CS/OJPs/CL NPs was slow during the first 6 h in acidic medium (pH 3.0, simulating the pH of fed gastric fluids), but the release became faster in pH 7.4 buffer (simulating the pH of terminal ileum). Accordingly, they should have great potential for the oral administration of biologically active compounds.

We additionally examined the effect of enzymatic degradation of the NPs on OJPs release. The release profile of OJPs from CS/OJPs/CL NPs was investigated by exposing them to the release medium containing lysozyme and trypsin, respectively. In enzyme-free, pH 6.5 buffer, only 20% of incorporated OJPs was released from CS/OJPs/CL NPs after 12 h. However, in the same release medium containing lysozyme (1 mg/mL), about 70% of OJPs were released from the NPs. Similarly, OJPs released from CS/OJPs NPs were enhanced in the release medium containing lysozyme. As previously studied (Figure 4), CS/OJPs/CL and CS/OJPs NPs were highly sensitive to lysozyme degradation, resulting in enhancement of the OJPs release rate. In release medium containing trypsin (0.5 mg/mL), CS/OJPs/CL NPs exhibited faster OJPs release within 12 h compared to OJPs release in trypsin-free medium (47% vs. 20%). However, the release rate of OJPs from OJPs/CL NPs was not significantly enhanced by trypsin, suggesting that CL predominated the trypsin-triggered OJPs release from CS/OJPs/CL NPs.

Koo et al. reported that CS-coated and fucoxanthin-loaded CA NPs demonstrated better bio-accessibility of fucoxanthin under in vitro simulated digestion [51]. Our study shows that after incubation in SIF (pH 6.5) containing trypsin, the particle size of CS/OJPs/CL NPs increased significantly, and OJPs began to release at 12 h. Indicating the NPs can have the ability to protect drugs or proteins from fast enzymatic degradation by trypsin in intestinal juice and could release the cargo by CL-degradation-induced disintegration of NPs after long-term digestion.

### 3.5. In Vitro Antioxidant Activity

**Figure 6:**
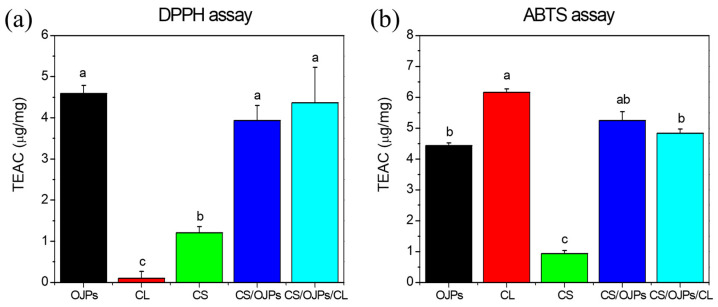
Antioxidant activity of CS/OJPs and CS/OJPs/CL NPs: (**a**) DPPH and (**b**) ABTS radicals scavenging activities. The different lowercase letters represent statistically significant differences (*p* < 0.05).

DPPH and ABTS free radicals scavenging assays are commonly used to measure the antioxidant activity of naturally occurring compounds. To understand the antioxidant activity of CS/OJPs and CS/OJPs/CL NPs and their compositions (polysaccharides and protein hydrolysis), the DPPH radical scavenging activities of CS, CL, OJPs and the NPs were measured (Figure 6). For the scavenging capacity against DPPH radical of free OJPs, CL and CS were 4.6, 0.1 and 1.2 μg/mg trolox equivalent antioxidant capacity (TEAC). OJPs shows high scavenging capacity against DPPH, which is consistent with previous studies reporting the antioxidant activity of Ophiopogon japonicus extract [62]. CS has good DPPH scavenging ability (1.2 μg/mg TEAC) because of its hydrogen-donating ability but the DPPH scavenging capacity of CL was poor. The DPPH scavenging capacity of CS/OJPs and CS/OJPs/CL NPs were 3.94 and 4.36 μg/mg TEAC, indicating that OJPs and CS had major roles in the DPPH scavenging activity of CS/OJPs/CL NPs (Figure 6a). The scavenging capacity against ABTS radical of CS was weak (0.93 μg/mg TEAC) while CL and OJPs have stronger ABTS radical scavenging ability (6.2 and 4.4 μg/mg TEAC) than CS (Figure 6b). Chang et al. has reported the antioxidant activity of CL with well-established antioxidants [63]. There was no difference in ABTS scavenging capacity between CS/OJPs and CS/OJPs/CL NPs (5.25 and 4.84 μg/mg TEAC) (Figure 6b). The study showed that the encapsulation of astaxanthin (ASTX) in stearic acid-CS conjugates/CA NPs significantly enhanced the antioxidant activity of ASTX against ABTS radicals [47]. Similarly, sinapic acid-CS/CA and gallic acid-CS/CA/oxidized dextran NPs greatly enhanced the antioxidant activity and sustainability of black rice anthocyanins and curcumin against DPPH and ABTS radicals [53,55]. However, our study shows that CS/OJPs/CL NPs did not significantly enhance the DPPH and ABTS radical scavenging activity of OJPs. This may be due to the poor dispersibility of free ASTX and curcumin in water, thus limiting its reaction with DPPH and ABTS radicals. In contrast, OJP is readily soluble in water and thus exhibits good free radical scavenging activity.

### 3.6. Phagocytic Uptake and Cytotoxicity of Nanoparticles

**Figure 7:**
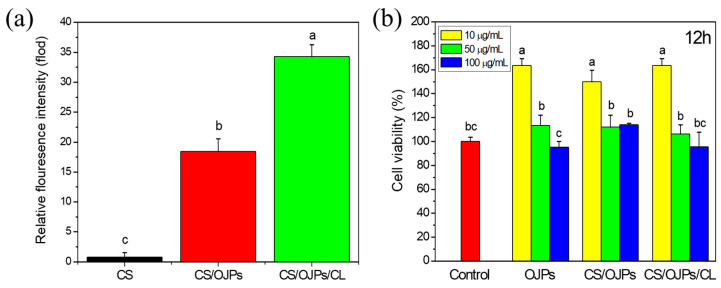
Phagocytic uptake and cytotoxicity: (**a**) cellular uptake CS/OJPs and CS/OJPs/CL NPs by RAW264.7 cells and (**b**) cell viability of RAW264.7 cells incubated together with CS/OJPs and CS/OJPs/CL NPs. The different lowercase letters represent statistically significant differences (*p* < 0.05).

Mucoadhesive chitosan particles and hydrogels have been used for delivery of drugs and active compounds to treat chronic inflammation of intestines, including ulcerative colitis and inflammatory bowel diseases (IBD) [64,65]. Pro-inflammatory cytokines produced predominantly by activated macrophages are involved in the development of the diseases. Figure 7a shows the cellular uptake of CS, CS/OJPs and CS/OJPs/CL NPs by macrophages. Phagocytosis uptake of nanoparticles generally favors positive zeta potential and larger particles [66,67]. CS/OJPs and CS/OJPs/CL NPs have similar average particle size (195.5 nm vs. 198.1 nm) and zeta potential (1.09 mV vs. 0.56 mV). However, CS/OJPs/CL NPs were more efficiently taken up by macrophages than CS/OJPs NPs. This might be attributed to the plasma membrane penetration ability of CL [68].

Cell viability of the macrophages incubated together with CS/OJPs and CS/OJPs/CL NPs were determined by MTT assay to evaluate the safety of the NPs. As shown in Figure 7b, the cell viability of all the formulations (OJPs, CS/OJPs and CS/OJPs/CL NPs) were higher than 95% with OJPs equivalent up to 100 μg/mL (Figure 7b). Interestingly, OJPs, CS/OJPs, and CS/OJPs/CL NPs resulted in 1.5–1.6-fold higher cell viability than the control at a low dose of 10 μg/mL OJPs equivalent, which could be attributed to the immunoregulatory activity of OJPs [8]. These findings suggested that CS/OJPs and CS/OJPs/CL NPs were non-cytotoxic to the macrophage cells.

### 3.7. Protective Effect against Ni^2+^-Induced Cytotoxicity and LPS-Induced Inflammation

**Figure 8:**
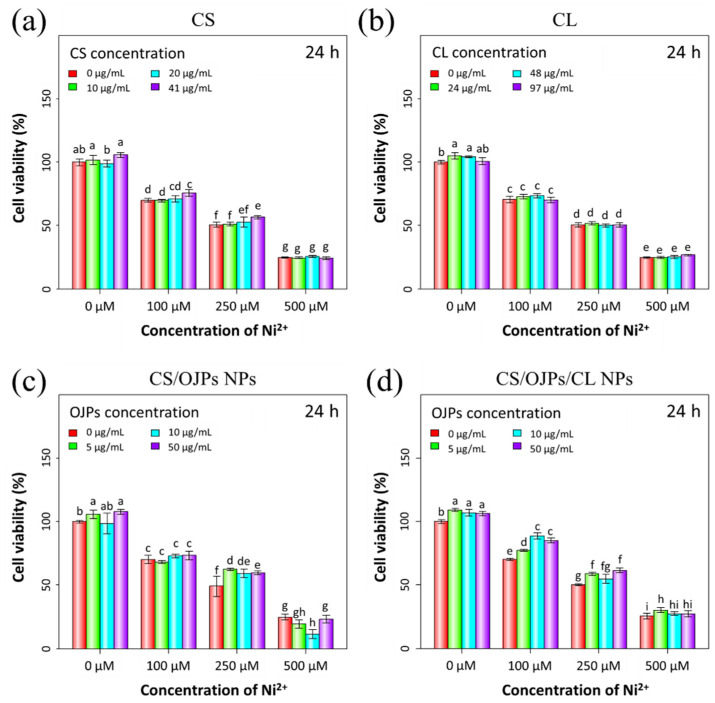
Inhibitory effect of CS/OJPs and CS/OJPs/CL NPs against Ni^2+^-induced cytotoxicity in RAW264.7 cells. Raw materials (**a**,**b**) and nanoparticles (**c**,**d**). The different lowercase letters represent statistically significant differences (*p* < 0.05).

Nickel has cytotoxicity towards macrophages and can induce allergic contact hypersensitivity [69]. In this work, Ni^2+^-induced cytotoxicity effect in macrophages was examined by measuring cell viability of macrophages incubated together with Ni^2+^. For this, CS/OJPs and CS/OJPs/CL NPs were added to the above cells/Ni^2+^ cultures to investigate the protective effect of the NPs on Ni^2+^-induced cytotoxicity in RAW264.7 cells. Significant cell viability decrease was observed after exposure to 100 and 250 μM of Ni^2+^ (Figure 8c). CS/OJPs and CS/OJPs/CL NPs attenuated Ni^2+^-induced cytotoxicity in RAW264.7 cells and thus increased their viability (Figure 8c,d). Our previous study has observed that OJPs can protect RAW264.7 cells from the Ni^2+^-induced cytotoxicity effect by enhancing cell viability from 50% to 57% and from 75% to 80% under 50 μg/mL [56]. By comparison, CS/OJPs/CL NPs can improve to 61% and 91% from 50% and 75% cell viability, respectively, to protect macrophages from Ni^2+^-induced cytotoxicity (Figure 8). This is most likely due to the more efficient cellular uptake of OJP delivered via the NPs (Figure 7a).

**Figure 9:**
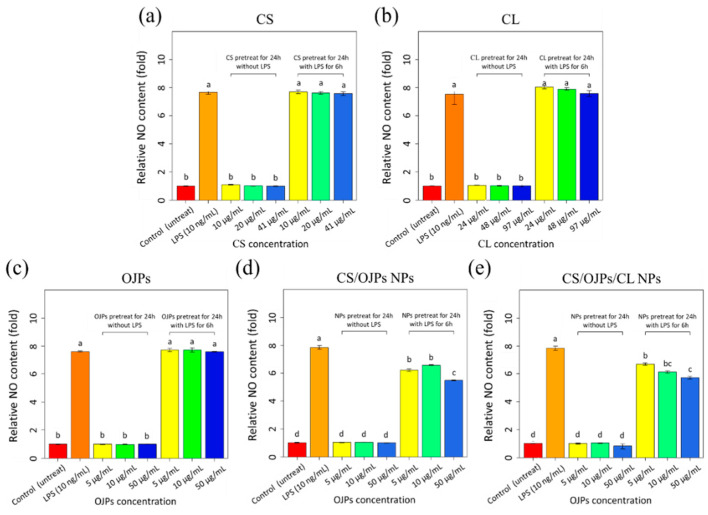
Inhibitory effect of CS/OJPs and CS/OJPs/CL NPs against LPS-induced NO production in RAW264.7 cells. Raw materials (**a**–**c**) and nanoparticles (**d**,**e**). The different lowercase letters represent statistically significant differences (*p* < 0.05).

We further tested the anti-inflammatory effects of NPs in LPS-stimulated macrophage model. As shown in Figure 9, the levels of pro-inflammatory signal and nitric oxide (NO) were increased 7.8-fold in LPS-activated RAW264.7 cells compared to non-activated controls. The NO production of CS/OJPs and CS/OJPs/CL NPs-treated cells decreased as the NPs concentration increased, significantly lower than the LPS-activated group with a 30% decrease (Figure 9d,e). However, no statistical differences of NO levels were observed between CS/OJPs and CS/OJPs/CL NPs-treated groups. CS/OJPs and CS/OJPs/CL NPs more effectively suppressed LPS-induced NO production in macrophage than free OJPs (Figure 9c), indicating that the NPs indeed help to enhance the OJPs-involved anti-inflammatory effect. This may be attributed to the fact that CS/OJPs and CS/OJPs/CL NPs could enhance cellular uptake of OJPs, thereby more effectively reducing NO levels in LPS-stimulated macrophages.

## 4. Conclusions

In summary, the polysaccharides/protein hydrolysate complex nanoparticles were successfully fabricated using CS, OJPs, and CL for the purpose of oral delivery of a group of antioxidant and anti-inflammatory active polysaccharides, OJPs, and the optimal compositions for preparation of CS/OJPs and CS/OJPs/CL NPs were investigated. The particle size of the optimized NPs is close to 200 nm, and the encapsulation efficiency of OJPs is 88.1 ± 0.2%. The NPs exhibited pH-responsive properties with positive zeta potential at pH lower than 6.5, revealing that the predominant cover on the particles’ surface is CS. In vitro drug release studies demonstrated that the NPs were able to control the release of OJPs in release media simulating the pH of the digestive tract, and that the OJPs release rates varied at different pH values and were accelerated by enzymatic degradation. OJPs delivered by the NPs showed excellent macrophage cellular internalization efficiency, thereby improving the anti-inflammatory ability of OJPs, like giving a more than 30% decrease of LPS-induced NO release. Moreover, the NPs-delivered OJPs increased, by 3.2-fold, their inhibition of nickel-induced toxicity to macrophages compared to free OJPs.

The findings in the current study demonstrated that self-assembled CS/OJPs/CL NPs can be easily prepared by the PEC method and possess multiple functionalities, such as pH/enzyme-responsive controlled release properties. These properties will be beneficial for reducing the degradation of OJPs and increase their bioavailability. However, much remains to be explored in the future, such as in vivo animal studies to illustrate the permeation efficiency in the mucus layer and small intestine, and the oral absorption efficiency of the prepared nanoparticles.

## Figures and Tables

**Table 1:** Mean particle size (hydrodynamic diameter), zeta potentials, and OJPs encapsulation efficiency of CS/OJPs NPs and CS/OJPs/CL NPs.

	Mean Particle Size	Zeta Potential	Encapsulation Efficiency
	(nm)	(mV)	(%)
**CS/OJPs NPs**	195.5 ± 4.6	1.1 ± 0.3 (pH 6.5)	78.6 ± 1.4
		5.8 ± 1.1 (pH 6.0)	
		12.9 ± 2.3 (pH 5.0)	
**CS/OJPs/CL NPs**	198.1 ± 6.8	0.6 ± 0.1 (pH 6.5)	88.1 ± 0.2
		5.5 ± 1.3 (pH 6.0)	
		13.6 ± 2.5 (pH 5.0)	

## Data Availability

Not applicable.
